# Long-Term Effects of Antiviral Therapy in Patients with Chronic Hepatitis C

**DOI:** 10.1155/2010/562578

**Published:** 2010-09-27

**Authors:** Tatehiro Kagawa, Emmet B. Keeffe

**Affiliations:** ^1^Department of Gastroenterology, Tokai University School of Medicine, Shimokasuya 143, Isehara 259-1193, Japan; ^2^Division of Gastroenterology and Hepatology, Department of Medicine, Stanford University Medical Center, Palo Alto, CA 94304-1509, USA

## Abstract

Chronic hepatitis C is a major cause of chronic liver disease globally, and the natural history of progression may lead to cirrhosis with liver failure, hepatocellular carcinoma, and premature liver-related death. Emerging data demonstrates that interferon-based therapy, particularly among those achieving a sustained virologic response (SVR), is associated with long-term persistence of SVR, improved fibrosis and inflammation scores, reduced incidence of hepatocellular carcinoma, and prolonged life expectancy. This reduction in the rate of progression has also been demonstrated in patients with chronic hepatitis C and cirrhosis in some but not all studies. The majority of these results are reported with standard interferon therapy, and long-term results of peginterferon plus ribavirin therapy with a higher likelihood of SVR should have a yet greater impact on the population of treated patients. The impact on slowing progression is greatest in patients with an SVR, less in relapsers, and equivocal in nonresponders. Thus, the natural history of chronic hepatitis C after completion of antiviral therapy is favorable with achievement of an SVR, although further data are needed to determine the likely incremental impact of peginterferon plus ribavirin, late long-term effects of therapy, and the benefit of treatment in patients with advanced hepatic fibrosis.

## 1. Introduction

Hepatitis C virus (HCV) is a leading cause of chronic liver disease and a major public health problem, with 130 to 170 million people infected worldwide [1]. Chronic HCV infection may result in serious sequelae, such as end-stage cirrhosis, hepatocellular carcinoma (HCC), need for liver transplantation, and premature death [2]. Treatment of HCV infection with interferon was first used successfully in 1986 [3], and interferon in its pegylated formulation in combination with ribavirin is the preferred therapy for the treatment of patients with acute or chronic HCV infection [4]. The current treatment of chronic hepatitis C is peginterferon alfa-2a or alfa-2b plus ribavirin, with approximately 55% to 65% of patients across all genotypes achieving a sustained virologic response (SVR) [5–7]. However, only 42% to 52% of patients with genotype 1 infection have an SVR after 48 weeks of combination therapy in pivotal trials [5–7]. SVR is traditionally defined as an undetectable serum HCV RNA 24 weeks after the discontinuation of therapy and is regarded as a “cure” [4], although an undetectable HCV RNA 12 weeks after completion of therapy appears to be equally as relevant for prediction of an SVR in patients treated with peginterferon plus ribavirin [8]. The primary goal of treatment of chronic hepatitis C is to prevent late liver-related morbidity and mortality, and measurement of SVR is the short-term surrogate used to predict the long-term efficacy of antiviral therapy. The purpose of this concise paper is to summarize current knowledge on the impact of antiviral therapy on persistence of SVR and long-term outcomes, including impact on hepatic fibrosis, incidence of HCC, and life expectancy. These data provide a rationale for the early use of antiviral therapy not only to achieve an SVR but also to favorably impact the long-term prognosis of chronic hepatitis C.

## 2. Sustained Virological Response Persists Long Term

The earliest study demonstrating that a sustained response to antiviral therapy was associated with long-term biochemical and virological responses as well as histologic improvement was reported by Marcellin and colleagues from France [9]. In this study of 80 patients who had a 6-month sustained biochemical and virologic response, mean followup of 4 years showed that 93% of patients had a persistently normal alanine aminotransferase (ALT) level, and 96% had undetectable serum HCV RNA. A comparison of hepatic histology before and 1 to 6.2 years after completion of interferon therapy showed improvement in 94% of patients, and HCV RNA was undetectable in the liver in 1 to 5 years after treatment in all 27 patients tested. In an analysis of 4 large trials in which 395 patients were followed after achieving an SVR with interferon alfa-2b with or without ribavirin, the actuarial likelihood of maintaining response after a mean 5-year followup was 99%  ±  1%, with overall 10 patients (2.5%) developing detectable HCV RNA and all within 2 years of followup [11]. Thus, this analysis of a large study database confirmed that late relapse is rare in patients who remain HCV RNA negative 24 weeks after completion of interferon-based therapy. Multiple other studies [10, 12–18], including one small study with a 10-year mean followup [19], showed that SVR predicts a high likelihood of long-term SVR (Table 1). One of these studies followed 344 patients for a median duration of 3.3 years (range, 0.5 to 18 years) after completion of interferon-based therapy with an SVR, and showed that serum HCV RNA remained undetectable in 1300 samples, indicating that none had a relapse with up to 18 years of followup [16]. It is unclear if late detection of serum HCV RNA after an SVR in a small number of patients represents true relapse, especially if the detection occurs only once or is intermittent and with use of a very sensitive assay. Although additional followup studies may provide further clarification of this distinction, an SVR appears for now to be durable and an accurate reflection of a cure.

## 3. Natural History of Chronic Hepatitis C

The progression of HCV infection is relatively slow, and the risk of developing cirrhosis ranges from 4%–25% in infected persons over 20–30 years of followup [4, 26–28]. Older age at the time of HCV infection, male gender, obesity, immunosuppression, and heavy alcohol intake are associated with a more rapid progression rate [28–32]. The annual rate of developing HCC in the presence of cirrhosis ranges from 1%–3% in Western countries to 5%–7% in Japan [33–38]. The higher proportion of elderly patients infected with HCV in Japan may explain the higher incidence rate in this country, since older age accelerates the development of HCC [39]. Hepatic decompensation of cirrhosis occurs at a rate of 3% to 4% per year [33, 34].

## 4. Antiviral Therapy Improves Hepatic Fibrosis and Inflammation

Changes in histologic findings following a course of interferon therapy have been studied extensively. Shiratori and colleagues [40] analyzed paired liver biopsies of 593 patients; 487 received interferon monotherapy for no longer than 6 months and 106 were untreated. Fibrosis regressed at a rate of 0.28 Metavir units per year in those who achieved an SVR. A meta-analysis of data from 1,013 patients from 3 large randomized trials [41–43] demonstrated that peginterferon treatment reduced inflammation and fibrosis in patients with an SVR or who relapsed, but not in nonresponders [44]. This improvement was more prominent with use of peginterferon rather than interferon. Significant improvement has also been shown after use of peginterferon and ribavirin combination therapy, with achievement of SVR [45]. Even in patients with advanced fibrosis or cirrhosis, inflammation and fibrosis were improved with interferon monotherapy, peginterferon monotherapy, or peginterferon plus ribavirin combination therapy [44–46]. In a pooled set of data from 3,010 naïve hepatitis C patients with pretreatment and posttreatment biopsies form 4 randomized trials of 10 different regimens using combinations of standard interferon alfa-2b, peginterferon alfa-2b and ribavirin, it was shown that there was a 39% to 73% improvement in necrosis and inflammation [45]. In addition, all regimens significantly reduced fibrosis progression rates in comparison to rates before treatment, with the best results noted with peginterferon plus ribavirin. In particular, cirrhosis was reversed in 49% of patients with baseline cirrhosis. Six factors were associated with the absence of significant fibrosis after treatment: baseline fibrosis stage, SVR, age <40 years, body mass index <27 kg/m^2^, no or minimal baseline activity, and HCV RNA level <3.5 million copies/mL [45]. In contrast to the study of Cammà et al. [44], the analysis of Poynard and colleagues showed slowing of the natural progression of fibrosis in nonresponders, as well as those with an SVR and relapsers [45]. A limitation of both of these studies is the relatively short time between paired biopsies, that is, before treatment and 24 week after completion of therapy.

In summary, studies of antiviral therapy with interferon monotherapy, peginterferon monotherapy, or peginterferon plus ribavirin combination therapy demonstrate an improvement in inflammation and fibrosis in patients with an SVR, to a lesser extent in relapsers, and uncertain benefit in nonresponders.

## 5. Antiviral Therapy Is Associated with a Reduced Incidence of Hepatocellular Carcinoma

The Inhibition of Hepatocarcinogenesis by Interferon Therapy (IHIT) Study Group in Japan has been studying the development of HCC in approximately 3,000 Japanese patients with chronic hepatitis C and has demonstrated that interferon therapy reduces the risk of HCC by half including down to one-fifth in biochemical or virological responders, compared with untreated patients [37]. The preventive effect of interferon on development of HCC was also reported by Imai et al. [39], who conducted a retrospective study of 419 patients receiving interferon monotherapy for 6 months and compared the incidence rate of HCC with untreated controls. The relative risk (RR) for HCC was 0.06 (*P* = .007) in patients with an SVR, and 0.51 (*P* = .15) in relapsers, and 0.95 (*P* > .2) in nonresponders, indicating that only an SVR was associated with a reduced risk of HCC. In contrast, another study demonstrated that the incidence of HCC was decreased not only in patients with an SVR, but also in relapsers when compared with nonresponders [47]. Compatible with the studies cited above, the IHIT Study Group has also demonstrated reduced inflammation and fibrosis in patients with an SVR [40]. However, it should be noted that a reduction in the risk of HCC does not necessarily indicate improvement in overall survival, and interferon is less effective in patients with cirrhosis. In addition, cirrhotic patients tend to be older, and liver-unrelated mortality may be significant and obscure any potential benefit of interferon therapy.

## 6. Life Expectancy Is Prolonged with Interferon-Based Therapy

It would be expected that long-term durability of an SVR, improvement in fibrosis and inflammation, and a reduced incidence of HCC would translate into a prolonged life expectancy. In fact, recent studies with sufficiently long followup are now reporting this ultimate end-point of antiviral therapy [36, 48–50]. In a retrospective cohort study of 7 university hospitals and 1 regional core hospital in Japan, 2,889 patients with biopsies, including 2,430 patients receiving interferon and 459 untreated patients were analyzed [49]. Compared with the general population, overall mortality was high among untreated patients but not among the treated patients. In the interferon-treated patients, the risk of liver-related death was reduced compared with untreated patients, while the risk of liver-unrelated death remained unchanged. In another study of 459 patients followed for a mean of 8.2 years, multivariate regression analysis revealed that interferon treatment decreased the risk ratio for overall death and liver-related death, particularly in patients with an SVR [48]. Once again, interferon showed no association with liver-unrelated death. Another retrospective cohort study of 2,954 patients, including 2,698 who were treated and 256 who were untreated, showed similar results [50]. Over a mean 6-year followup, interferon therapy improved survival in chronic hepatitis C patients showing a biochemical as well as a virological response by preventing liver-related deaths. Finally, a prospective cohort 6.8-year followup study of 345 patients with cirrhosis, of whom 271 were treated and 74 not treated, showed that interferon inhibited the development of HCC and also improved survival [36]. In contrast to the above findings, an Australian study demonstrated that IFN treatment reduced liver complications, but the beneficial effect of achieving an SVR was marginal (*P* < .058) in a multivariate analysis [51].

## 7. Impact of Antiviral Therapy in Patients with Chronic Hepatitis C and Cirrhosis

What about the impact of antiviral therapy in patients with chronic hepatitis C and cirrhosis? Shiratori et al. [36] conducted a prospective study on 345 cirrhotic patients, of whom 271 received interferon monotherapy for a median of 6.5 months, evaluating the cumulative incidence of HCC and survival relative to 74 untreated patients; SVR was attained in 15% of these patients. In patients who achieved an SVR, the age-adjusted hazard ratio (HR) for developing HCC and death was significantly reduced to 0.31 (*P* < .001) and 0.05 (*P* = .003), respectively, whereas the outcomes for those who did not attain an SVR were not significantly different from the control group. Bruno et al. [52] performed a retrospective cohort study of 920 cirrhotic patients treated with interferon monotherapy for 1 year, which resulted in an SVR rate of 13.5%. Failure to achieve an SVR had a higher risk of liver-related complications, HCC, and liver-related mortality. Thirty-three percent of patients achieved an SVR after peginterferon plus ribavirin combination therapy and had better outcomes than those who did not attain an SVR [53]. Multivariate analysis revealed that a failure to achieve an SVR was associated with a higher risk of liver-related complications, HCC, and liver-related mortality compared to those who achieved an SVR. 

However, several studies have demonstrated no beneficial effect of IFN therapy on the prognosis of cirrhotic patients [33, 54, 55]. A lower SVR rate (4%–9%) or shorter followup period (median, 2.1 years) in these studies may have underestimated the benefit of interferon therapy. A recently published meta-analysis demonstrated that antiviral treatment was associated with a reduced risk of HCC in patients who attained an SVR, compared with nonresponders; the best outcomes were seen in patients treated with ribavirin-based regimes, which confirms the results of other meta-analyses [56–58]. The attainment of SVR also demonstrated prevention of the development of esophageal varices [34].

There have been case reports [59] and long-term followup studies that have shown the development of HCC in patients with advanced hepatic fibrosis after the achievement of an SVR [17, 36, 52, 55, 60]. In one followup study, the two patients who developed HCC were diagnosed 5.8 and 7.3 years after having achieved an SVR. These observations underscore the continued risk of HCC and need for ongoing surveillance with imaging and alpha-fetoprotein testing in patients with chronic hepatitis C and advanced hepatic fibrosis, even after an SVR.

In summary, multiple studies have shown that the achievement of an SVR with antiviral therapy reduces, but does not eliminate, the incidence of HCC and decreases liver-related complications and liver-related mortality in patients with chronic hepatitis C, and in some studies also in cirrhotics. However, the effect of antiviral therapy is controversial in patients who were responders during treatment and subsequently relapsed after treatment and probably has limited benefit in nonresponders.

## 8. Preventive Effect of Interferon on Recurrence of Tumor after Treatment of HCC

To evaluate effects of interferon treatment on HCC recurrence after resection of HCC, studies were performed in which patients were randomized to receive interferon treatment or to remain untreated [61, 62]. Patients in the interferon-treated group received interferon alpha for approximately 2 years, but only 2 patients (13%) achieved an SVR. HCC recurrence was observed in 33% of the interferon-treated group and in 80% of the control group. The recurrence rate was significantly lower and the cumulative survival rate significantly higher in the interferon group than in the control group [61, 62]. In another randomized controlled study, 74 patients with cirrhosis and low HCV RNA loads were assigned to receive interferon for 1 year (*n* = 49) or to an untreated control group (*n* = 25) after complete ablation of HCC by percutaneous ethanol injection therapy [63]. SVR was achieved in 29% of patients. Interferon treatment seemed to suppress the rate of a second or third recurrence of HCC, but not a first recurrence, and improve the survival rate. Several studies, including one meta-analysis with one exception, indicate that the achievement of SVR appears to prevent the recurrence of HCC [64–69]. However, as virtually all of these studies were conducted in Japan, a definitive conclusion should await further reports from other regions of the world.

## 9. Impact of Maintenance Therapy on Outcomes of Chronic Hepatitis C with Advanced Fibrosis

An earlier randomized controlled trial revealed that long-term low-dose interferon treatment for patients who did not obtain an SVR with previous standard interferon-based therapy improved liver histology [20] (Table 2). In addition, Nishiguchi et al. [70, 71] demonstrated that interferon monotherapy for 12 to 24 weeks reduced the occurrence of HCC and disease progression. Although these studies included a small number of patients, the results encouraged clinicians to perform large studies to confirm the impact of maintenance therapy for chronic hepatitis C with advanced hepatic fibrosis.

The HALT-C study enrolled 1,050 patients with bridging fibrosis or cirrhosis (defined as an Ishak fibrosis score of 3 or more) who had not responded to a previous 6- or 12-month course of therapy (lead-in phase) with peginterferon and ribavirin [22, 72]. These patients were randomized to receive either no therapy or peginterferon alfa-2a at a dose of 90 *μ*g per week for 3.5 years. Although serum aminotransferase levels, serum HCV RNA levels, and necroinflammatory scores decreased significantly with therapy, the rate of primary outcome (death, HCC, hepatic decompensation, or an increase in the Ishak score of 2 or more points) was similar in the treatment and control groups (34.1% and 33.8%, respectively), confirming the results of a previously reported randomized, controlled trial with a relatively small number of patients [21].

In the HALT-C study, viral suppression of ≥4 log_10_ during the lead-in phase of therapy was associated with a significant reduction in clinical outcomes, although a significant difference was not observed between the treatment and control groups [73]. Unexpectedly, viral suppression of ≥4 log_10_ during the phase of maintenance therapy did not result in an improvement in clinical outcomes. A decrease in serum HCV RNA levels might improve the clinical outcome, even if short-term and transient; thus viral suppression during the lead-in phase may have obscured the beneficial effect of maintenance therapy. The Nishiguchi study tested interferon-naïve patients, which may have accentuated the beneficial effects of interferon [70]. Sixteen percent of treated patients achieved an SVR in this study, which is unlikely in studies enrolling previous nonresponders.

The results from two other large randomized trials are currently unpublished. In the COPILOT study, patients with an Ishak fibrosis score of 3 or more who were nonresponders to interferon therapy were randomized to receive either peginterferon alfa-2b, at a dose of 0.5 *μ*g/kg weekly, or colchicine. This maintenance therapy significantly retarded the development of varies and prevented variceal bleeding, but did not affect overall outcome [23, 24]. The EPIC^3^ study also revealed that maintenance therapy was not superior to control except in reducing clinical events in patients with esophageal varices [25]. Finally, a recent meta-analysis assessing the role of the maintenance interferon therapy in nonresponders did not reduce the incidence of HCC (RR: 0.58; 95% CI: 0.33–1.03) [56].

In summary, there are insufficient data to recommend maintenance therapy for patients who did not respond to previous interferon treatment. In the HALT-C study, 3.5 years of low-dose peginterferon improved histologic necroinflammatory scores, but did not reduce the rate of disease progression. Liver histology, especially fibrosis, is closely associated with prognosis [37, 48, 74, 75]. Therefore, further followup of patients in the HALT-C trial may confirm the inhibitory effect of the maintenance therapy on the progression of liver disease.

## 10. Conclusions

The long-term benefit of antiviral therapy, including reduction in hepatic fibrosis, lower incidence of HCC, and prolonged life expectancy, appears to be limited primarily to patients able to achieve an SVR. A recent systematic review showed that health-related quality-of-life was also improved and that antiviral treatment is reasonably cost effective in treatment-naïve patients as well as relapsers and nonresponders [76]. Therefore, clinicians should aim to treat with antiviral therapy, with the goal of achieving an SVR early in the natural history of chronic hepatitis C. Direct acting antiviral (DAA) agents are expected to provide new treatment options for management of chronic hepatitis C in the near future. Telaprevir, an HCV NS3 protease inhibitor, in combination with peginterferon and ribavirin, induced SVR in approximately 70% of treatment-naïve genotype 1 patients and in 51% of patients who failed previous peginterferon plus ribavirin therapy [77–79]. The increase in SVR rate using DAA agents may provide a long-term benefit to a wider variety of patients, including nonresponders to peginterferon and ribavirin therapy, and further improve the long-term outcomes of therapy including slowed fibrosis progression, reduced incidence of HCC, and prolonged life expectancy.

## Figures and Tables

**Table 1:** Durability of undetectable serum HCV RNA after SVR.

Author, year of publication (reference)	Patients no.	Followup period years	Detectable HCV RNA no. (%)
Marcellin et al. [9]	80	4 (mean)	3 (4)
Reichard et al. [10]	26	5.4 (mean)	2 (8)
McHutchison et al. [11]	395; 151	5 (mean)	10 (2.5%); 2 (1.3)^1^
Veldt et al. [12]	286	Up to 4.9	12 (4)
Formann et al. [13]	187	2.4 (median)	0 (0)
Desmond et al. [14]	147	2.3 (mean)	1 (0.7)
Lindsay et al. [15]	366	4.8 (mean)	4 (1)
Maylin et al. [16]	344	3.3 (mean)	0 (0)
George et al. [17]	147	5.4 (median)	0 (0); 9 (6)^2^
Kim et al. [18]	73	Not reported	8 (11); 1 (1.4)^3^

Patients in the above studies who were followed after an SVR are heterogeneous and include those with all genotypes and various treatment regimens, including interferon monotherapy, interferon plus ribavirin and peginterferon plus ribavirin, and various treatment durations for 24 or 48 weeks; patients were generally naïve to prior therapy, but relapsers were included in some studies.

^1^395 patients with an SVR and participating in 4 studies were followed, including naïve and relapsed patients, and patients were treated with interferon monotherapy or interferon plus ribavirin for 24 or 48 weeks; of these 395 patients, a subset of 151 patients were naïve and received interferon plus ribavirin for 48 weeks.

^2^No patient had detectable HCV RNA using PCR (sensitivity = 29 IU/mL); 9 patients had HCV RNA detectable by TMA (sensitivity = 5.3 IU/mL) on one sample (mean of 4 samples from the 9 patients), but all other samples of these 9 patients were negative by TMA.

^3^HCV RNA was detectable by qualitative PCR in 8 patients, but only one patient had persistent viremia.

**Table 2:** Randomized, controlled trials to evaluate the effect of maintenance therapy on the progression of HCV-related chronic liver diseases.

Author (study name) [reference number]	Year	Patients	Patient no. (treatment/ control)	IFN regimen	Treatment duration (year)	Control	Preventing effect				
							Liver histology		Disease progression	HCC incidence	Compensation-free survival
							Inflammation	Fibrosis			
Shiffman et al. [20]	1999	Chronic hepatitis (a)	27/26	IFN alfa-2b 3 MU 3 times/wk	2	observation	Yes	Yes	N/A (b)	N/A	N/A

Fartoux et al. [21]	2007	Cirrhosis (c)	51/51	IFN alfa-2a 3 MU 3 times/wk	2	observation	N/A	N/A	No	No	No

Di Bisceglie et al. [22] (HALT-C)	2008	Bridging fibrosis or cirrhosis (d)	517/533	PegIFN alfa-2a 90 *μ*g/wk	3.5	observation	Yes	No	No	No	No

Cardenas et al. [23] and Afdhal et al. [24] (COPILOT)	2009 (e)	Advanced fibrosis or cirrhosis (f)	282/266	PegIFN alfa-2b 0.5 **μ**g/kg/wk	4	Colchicines 1.2 mg/day	N/A	N/A	Yes (reduced variceal bleeding)	No	No (g)

Bruix et al. (EPIC^3^) [25]	2009 (e)	Cirrhosis (h)	631 (total)	PegIFN alfa-2b 0.5 **μ**g/kg/wk	3	observation	N/A	N/A	No (i)	N/A	No

(a) The patients of both arms received 6-month course of IFN monotherapy before randomization.

(b) N/A = not available.

(c) 36% and 44% of patients previously failed to respond to IFN monotherapy and IFN/ribavirin combination therapy, respectively.

(d) Patients did not have response to 6- or 12-month lead-in phase peginterferon plus ribavirin therapy.

(e) Only abstract is available.

(f) Patients did not have response to the previous therapy.

(g) Event-free survival was better only in patients with portal hypertension.

(h) Patients did not have response to the previous interferon plus ribavirin therapy.

(i) Clinical events were observed less frequently in the treated group in patients with baseline esophageal varices.
